# Report on “Axiomatizing Conditional Normative Reasoning”

**DOI:** 10.1007/s13218-024-00832-1

**Published:** 2024-03-06

**Authors:** Xavier Parent

**Affiliations:** TU Wien, Favoritenstrasse 9, 1040 Wien, Austria

**Keywords:** Conditional obligation, Betterness, Axiomatization, Automated reasoning, First-order reasoning

## Abstract

This is a report on the project “Axiomatizing Conditional Normative Reasoning” (ANCoR, M 3240-N) funded by the Austrian Science Fund (FWF). The project aims to deepen our understanding of conditional normative reasoning by providing an axiomatic study of it at the propositional but also first-order level. The focus is on a particular framework, the so-called preference-based logic for conditional obligation, whose main strength has to do with the treatment of contrary-to-duty reasoning and reasoning about exceptions. The project considers not only the meta-theory of this family of logics but also its mechanization.

This is a report on the (still on-going) project “Axiomatizing Conditional Normative Reasoning (ANCoR)” funded by the Austrian Science Fund (FWF) [M 3240-N].

I lead the project as the Principal Investigator (P.I.). This one is hosted by the Theory and Logic group at the Institute of Logic and Computation at the Technological University of Vienna, Austria. The main collaborators are the following persons—their input is gratefully acknowledged:

- A. Ciabattoni, host (Technological University of Vienna)—proof theory
- C. Benzmüller (Bamberg University)—automated theorem proving
- N. Olivetti (Aix-Marseille University, France)—proof theory and complexity
- D. Pichler (Technological University of Vienna)—semantics
- L. van der Torre (University of Luxembourg)—semantics

ANCoR aims to deepen our understanding of conditional normative reasoning by providing an axiomatic study of it at the propositional but also first-order level. The project investigates a particular deontic framework, the so-called preference-based logic for conditional obligation due to Hansson [1] and Lewis [2] among others. Two groups of frameworks have dominated the philosophical landscape. Those in the first are based on modal logic, while those in the second are rule-based systems. The preference-based logic for conditional obligation is an example of the first group of frameworks. Deontic logic faces significant challenges in representing contrary-to-duty obligations and exceptions. The preference-based logic for conditional obligation, among other approaches, has become a widely accepted standard for normative reasoning. This is attributed to its capacity to accommodate them.^1^ This framework has been part of the landscape for some time, but the study of its meta-theory is relatively new and many fundamental questions remain open, including a systematic axiomatic treatment. The language has a conditional obligation operator $\bigcirc (B/A)$ read as “If *A*, then *B* is obligatory” and viewed as a primitive construct. The semantics is in terms of preference models. In models of this sort, a binary (preference) relation $\succeq$ (“at least as good as”) ranks the possible worlds in terms of comparative goodness or betterness. $\bigcirc (B/A)$ holds, if all the best *A*-worlds are *B*-words.^2^

The project aims to deepen our understanding of conditional normative reasoning, by establishing a roadmap of the different systems that can be obtained based on two types of consideration. The first one is familiar from modal logic. It concerns the choice of the properties of the betterness relation in the models. The traditional ones are: reflexivity, transitivity, totality, and two versions of Lewis [2]’s limit assumption (which precludes the possibility of sets of worlds without a “best” element). When discussing conditionals, the second type of consideration to keep in mind is the definition of “best” that appears in the truth conditions for the conditional. There are two main definitions of “best”: optimality and maximality. This distinction is familiar from rational choice theory.^3^ ANCoR introduces a third and lesser-known concept of “best” called strong maximality. For *x* to qualify as an optimal element of *X* (set of worlds), it must be at least as good as every member of *X*. For *x* to count as a maximal element, no other element in *X* must be strictly better than it. Thus, while the optimal worlds are all equally good, the maximal ones are either equally good or incomparable. For *x* to be strongly maximal in *X*, no other worlds in *X* must be strictly better than any world equally as good as *x*. Due to Bradley [8], this rule of interpretation has yet to be studied in deontic logic. When one keeps the possibility of incomparability open, maximality is a more appropriate approach than optimality. When transitivity is dropped (as urged by moral philosophers and economists; see, e.g., [9]), strong maximality is more appropriate than maximality. The latter violates the seemingly plausible requirement that two equally good worlds should be equally best (choice-worthy).

Depending on what notion of “best” is used, one gets different truth conditions for the conditional obligation operator, but also different forms of the limit assumption. In the context of the ANCoR project, a systematic study of the correspondence between modal axioms and properties of the betterness relation under these different notions of “best” has been carried out. These correspondences are extracted by appropriate soundness and completeness theorems. Thus, “correspondence” is taken in the same (broad) sense that Hughes and Cresswell have in mind when they write:

“D, T, K4, KB [are] produced by adding a single axiom to K and [...] in each case the system turns out to be characterized by [sound and complete w.r.t.] the class of models in which [the accessibility relation] *R* satisfies a certain condition. When such a situation obtains—i.e. when a system K+$\alpha$ is characterized by the class of all models in which *R* satisfies a certain condition−we shall [...] say [...] that the wff $\alpha$ itself is characterized by that condition, or that the condition *corresponds* [their italics] to $\alpha$.” [10, p. 41]

Thus far, the project’s main results have had to do with (1) axiomatization for the non-transitive case, (2) complexity, and (3) automated reasoning. Future efforts will focus on the extension to (4) first-order deontic reasoning.

**(1) Axiomatization for the non-transitive case**

An overview of the correspondences is given in [11, 12]. The main breakthrough of the project is reported in [13]. It concerns the property of transitivity of the betterness relation and four candidate weakenings of it discussed in rational choice theory: quasi-transitivity [14], a-cyclicity, Suzumura consistency [15], and Fishburn [16]’s condition of an interval order. The transitivity of betterness is usually taken for granted in deontic logic. There is a call for understanding what happens when one lets it go or weakens it suitably. Early results were for the case where the betterness relation comes with the full panoply of the standard properties. As mentioned, the transitivity of betterness has been criticized by moral philosophers and economists.

The first group of findings concerns transitivity and its first three weakenings (quasi-transitivity, a-cyclicity and Suzumura consistency). It has been discovered that they have no syntactical counterpart. Hence they have a less important role to play than one would have thought. Indeed, the logic remains the same whether or not one introduces these conditions. This is shown with reference to a series of systems of increasing strength: Åqvist [17]’s systems **E** and **F**; and system **F**+(CM) [18]. **E** is the weakest system among those studied in the project. No properties are assumed of the betterness relation. **E** is characterized by (is sound and complete w.r.t.) the class of all preference models. **F** rules out the possibility of conflicts between obligations. Its distinctive axiom is $\Diamond A\rightarrow \lnot (\bigcirc (B/A) \wedge \bigcirc (\lnot B/A))$, where $\Diamond$ is read as “It is possible that”. The Kantian principle “*ought* implies *can*”, $\bigcirc (B/A)\rightarrow (\Diamond A \rightarrow \Diamond (A\wedge B))$, becomes derivable. **F** is determined by the class of models in which the truth set of every satisfiable formula has a “best” element. This first version of Lewis’s limit assumption is called limitedness. **F**+(CM) is obtained by supplementing **F** with the principle of cautious monotony (CM). It warrants the move from $\bigcirc (B/A)$ and $\bigcirc (C/A)$ to $\bigcirc (B/A\wedge C)$. This principle says that fulfilling an obligation does not affect our other obligations arising in the same context. **F**+(CM) is characterized by the class of models whose relation $\succeq$ meets a stronger version of Lewis’s limit assumption, called smoothness. It has been shown that each system is similarly characterized by the class of models whose relation $\succeq$ is in addition transitive, quasi-transitive, acyclic, or Suzumura consistent. This holds true for both maximality and strong maximality. As far as I know, these results have not been previously reported in the literature.

The second group of findings concerns the condition of an interval order. This condition allows for intransitivities of equal goodness (due to discrimination thresholds), and possesses a strong intuitive support. Its syntactical counterpart was pinpointed, in the form of the principle of disjunctive rationality (DR). The latter principle is familiar from the non-monotonic logic literature. It warrants the move from $\bigcirc (C/A\vee B)$ to either $\bigcirc (C/A)$ or $\bigcirc (C/B)$. Intuitively, if a disjunctive state of affairs triggers an obligation, then at least one disjunct triggers the obligation in question. **F**+(DR) has been shown to be weakly complete w.r.t. the class of finite models whose relation $\succeq$ is an interval order. The result holds for maximality and optimality and yields the finite model property and decidability. **F**+(DR) contains **F**+(CM) and is strictly weaker than Åqvist [17]’s well-known system **G**. This one is obtained by supplementing **F** with the principle of rational monotony (aka, Lewis [2]’s axiom CV). It has also been found that strong maximality boosts the logic from **F**+(DR) to **G**. In other words, under the rule of strong maximality, **G** is complete w.r.t. the class of models whose relation $\succeq$ is an interval order and limited. The finite model property also holds. As far as I know, these results have not been previously reported in the literature either.

Some authors reject the limit assumption and give a more complex semantic clause for the conditional obligation operator, like the $\exists \forall \exists$ rule (see, e.g., [11]). Only the case where the betterness relation is transitive, and the case where it is transitive and total are understood. The question of what happens in the absence of transitivity is still on the agenda.

ANCoR extended the scope of the inquiry in two directions: Gentzen-style proof theory and rule-based systems. I will just say a few words about the second. The chosen representative is the so-called input/output logic devised by Makinson and van der Torre [19–21]. The semantics is procedural and given in terms of a set of procedures yielding outputs for inputs. A systematic study of all the possible systems based on the choice of an input/output operation for obligation (and permission) has been carried out in [22]. The study goes beyond the original systems and includes new ones, allowing for better control of the detached obligations or permissions (modulo a built-in consistency check).

**(2) Complexity**

This work has benefited from a collaboration with A. Ciabattoni (TU Wien, Austria) and N. Olivetti (Aix-Marseille University, France) among others. We used their techniques, which involved delving into Gentzen-style proof theory. Analytic sequent calculi for **E** and **F** are given in [23] and [24], respectively. The property of analyticity implies that the cut rule (which corresponds to using lemmas in mathematics) is admissible. This property is particularly useful for automated reasoning. The study conducted in [23] reports a complexity result for **E**. This result shows that the validity problem in **E** (“Is the formula *A* valid?”) is Co-NP, and countermodels have a polynomial size, like in classical propositional logic. Therefore, conditional normative reasoning is not harder than ordinary (propositional) reasoning although it requires a more expressive language. This fact was not known before. It makes an essential use of the fact that the betterness ranking is not world-dependent, so all worlds agree on all conditional statements. The result echoes one previously obtained by Friedman and Halpern [25] in the related area of conditional logic. However, they work with models whose preference relation is a pre-order (is reflexive and transitive), and they use a more complex evaluation rule for the conditional, of the form $\exists \forall \exists$. The follow-up paper [24] reports a first complexity bound for system **F**. Deciding if a formula is a theorem of **F** is CoNEXP. An efficient method for extracting a counter-model from a failed proof attempt has been discovered for **E**, but not for **F**. The problem is with the limitedness condition; this one is not a frame condition.

**(3) Automated reasoning**

This work is carried out in collaboration with C. Benzmüller (Univ. of Bamberg). The shallow semantical embedding method developed over the years by his group has been successfully extended to the logic studied in this project. The basic idea consists in faithfully embedding a target logic (here Åqvist’s system **E**) into Higher-Order Logic (HOL), and then using an off-the-shelf HOL prover for automation. The faithfulness of the embedding is established in [26]. In general, the supported queries are:

- Proving a formula (via *Sledgehammer*)
- Disproving a formula or showing consistency by providing a model (via *Nitpick*).

The automatic verification of the correspondence between modal axioms and properties of the betterness relation in the models has been the main focus here. So far these correspondences have been established with pen and paper. We investigate the extent to which these can be verified by automated means. This is the first study of its kind. The implementation is written in Isabelle/HOL and is freely available on Github.^4^ We found that the provers are responsive (e.g., 7 ms to prove a validity), and that the choice of a backend matters: “Zipperposition” outperforms the others. The system gives answers, but also explanations. With respect to the first type of query, a reference to the required axioms and lemmas is returned. With respect to the second type of query, a (counter-)model is returned.

However, we found an asymmetry between conditional (deontic) logic and traditional modal logic. In the latter setting, the full equivalence between the property of the relation and the modal formula can be verified [27]. In the former setting only the direction “property $\Rightarrow$ axiom” is verified. To be more precise, what is verified is the fact that, if the property holds, then the axiom holds. What is not confirmed is the converse statement, that if the axiom holds then the property holds. The question as to whether the full equivalence can be established is a topic for future research.

The machinery has been applied to formalize and assess (some aspects of) Parfit [28]’s repugnant conclusion in population ethics. This work is still under review [29]; we find it promising.

**(4) First-order deontic reasoning**

This is an additional direction the ANCoR project is moving in. The propositional version of the aforementioned systems has been the primary and predominant focus so far.

First-order deontic reasoning has yet to be studied. It is commonly believed that the extension to the first-order case follows the same pattern as in other areas of modal logic. However, this is not entirely true. Recent evidence presented in [30] suggests that the theme of extensionality brings new and exciting challenges in a deontic context. This is based on one of Goble’s proposals, further documented in [31–33]. An operator is extensional if it allows substitution *salva veritate* of co-referential terms within its scope and intensional if it does not. It is commonly known that extensionality leads to the modal collapse, which states that every true statement is necessarily true and vice versa. There are reasons to believe that “ought” is extensional, unlike other modalities. The question arises as to whether it is possible to combine the extensionality and intensionality of different modal operators in the same semantics without creating the deontic collapse. The project answered this question within a particular system, namely system **F**. In the family of preference-based systems considered in this project, it is the weakest one in which the deontic collapse occurs. In the study [30], we develop in full detail a “perspectival” account of obligation, resolving the aforementioned problem. This solution is similar to the one proposed by Goble for Standard Deontic Logic (SDL) in the papers referenced above. We call the account “perspectival” because one always evaluates the content of an obligation in one world from the perspective of another, hence using some form of cross-world evaluation. The proposed framework uses the resources of two-dimensional modal logic (see, e.g., [34]) and allows for a more nuanced way of approaching first-order deontic principles. The paper [30] received the John-Jules Meyer Best Paper Award at the DEON 2023 conference.^5^

## Footnotes

1 For a fuller discussion, see Prakken and Sergot [3].

2 For further background on preference-based semantics for deontic logic, see Makinson [4] and Hilpinen and McNamara [5].

3 Most authors follow Herzberger [6] in using the terms “stringent” vs.“liberal” maximization for what (following Sen [7]) I call optimality vs. maximality. (I owe this observation to Sven Ove Hansson.)

4 http://logikey.org/ (file “DDLcube.thy” in sub-folder “Deontic-Logics/cube-ddl/”).

5 https://oraprdnt.uqtr.uquebec.ca/pls/public/gscw031?owa_no_site=7185&owa_no_fiche=15&owa_bottin=.
